# Evidence-based support for the all-hazards approach to emergency preparedness

**DOI:** 10.1186/2045-4015-1-40

**Published:** 2012-10-25

**Authors:** Bruria Adini, Avishay Goldberg, Robert Cohen, Daniel Laor, Yaron Bar-Dayan

**Affiliations:** 1 Emergency and Disaster Management Division, Ministry of Health, Tel Aviv, Israel; 2 PREPARED research center, Ben-Gurion University of the Negev, Beer-Sheva, Israel; 3 Department of Emergency Medicine, Leon and Mathilda Recanati School for Community Health Professions, Faculty of Health Sciences, Ben-Gurion University of the Negev, Beer-Sheva, Israel; 4 PREPARED Research Center and the Department of Emergency Medicine, The Leon and Mathilda Recanati School for Community Health Professions, Ben-Gurion University of the Negev, P.O.B 653, Beer-Sheva, 84105, Israel

**Keywords:** Emergency preparedness, Evidence-based, All-hazards approach, Evaluation, Mass casualty events

## Abstract

**Background:**

During the last decade there has been a need to respond and recover from various types of emergencies including mass casualty events (MCEs), mass toxicological/chemical events (MTEs), and biological events (pandemics and bio-terror agents). Effective emergency preparedness is more likely to be achieved if an all-hazards response plan is adopted.

**Objectives:**

To investigate if there is a relationship among hospitals' preparedness for various emergency scenarios, and whether components of one emergency scenario correlate with preparedness for other emergency scenarios.

**Methods:**

Emergency preparedness levels of all acute-care hospitals for MCEs, MTEs, and biological events were evaluated, utilizing a structured evaluation tool based on measurable parameters. Evaluations were made by professional experts in two phases: evaluation of standard operating procedures (SOPs) followed by a site visit. Relationships among total preparedness and different components' scores for various types of emergencies were analyzed.

**Results:**

Significant relationships were found among preparedness for different emergencies. Standard Operating Procedures (SOPs) for biological events correlated with preparedness for all investigated emergency scenarios. Strong correlations were found between training and drills with preparedness for all investigated emergency scenarios.

**Conclusions:**

Fundamental critical building blocks such as SOPs, training, and drill programs improve preparedness for different emergencies including MCEs, MTEs, and biological events, more than other building blocks, such as equipment or knowledge of personnel. SOPs are especially important in unfamiliar emergency scenarios. The findings support the adoption of an all-hazards approach to emergency preparedness.

## Background

During the last decade the need to respond to various emergencies such as natural disasters and technological and complex mass casualty events (MCEs) has increased [1]. While the nature of the events may differ significantly, preparedness for them appears to have much in common in terms of the knowledge and skills required [2,3]. Numerous mitigation programs have proven to be highly cost-effective in preparing for different types of crises [4].

Effective preparedness of hospitals for different hazards is more likely to be achieved if healthcare professionals adopt an all-hazards response plan that applies generic basic principles for managing different scenarios [5-8]. The all-hazards approach contends that emergency preparedness requires attention not just to specific types of hazards but also to actions that increase preparedness for all risks [7,8].

In view of these common components, the World Health Association (WHO) as well as other leaders in crisis management advocate the all-hazards approach as the recommended mechanism for emergency preparedness [9]. Nevertheless, the all-hazards policy has as yet not been fully adopted. Some experts support other programs such as utilization of risk assessment and reduction as a starting point for provision of goods and services based on needs assessment [10]. It has often been presented that capacity building programs focus on preparedness for a specific disaster; therefore, the legislation, administrative arrangements, and institutional structures are frequently created to respond to that scenario rather than to the common components that characterize different types of emergencies [11]. It has even been stated that despite lessons learned from disasters, increase in knowledge, and technological development, no shift in policy has been made with regard to crisis management [12].

Limited information is available with regard to what constitutes effective emergency preparedness; however, there is consensus that availability of a comprehensive Standard Operating Procedure (SOP), exercises and drills are important components in the preparedness process [13-15]. Implementation of realistic and well-run drills is a complex task requiring significant resources in terms of cost, manpower, and time commitment; thus the number and extent of drills are limited [16-18]. It would seem that there is much to be gained from identifying principles and knowledge that are common to all preparedness programs; ignoring these similarities and differences may hinder effective inter-agency collaboration [19].

The importance of preparing the medical system to deal with different emergencies while attempting to contain costs, suggests that it would be advisable to determine if common components can be identified. To date the relationship among preparedness for different types of emergency events has not been well investigated [8,20].

### Implementing an all-hazards approach in Israel

The Israeli healthcare system adopted and maintains an all-hazards approach to emergency management, basing its policy on preparedness for mass casualty events [2]. All hospitals are instructed to utilize similar principles in preparing for MCEs, mass toxicological events (MTEs), and biological events, and modify only components that are hazard-specific such as utilizing isolation facilities in biological events or decontamination of casualties in a toxicological event. The main components that are implemented as the result of this policy include designation of similar admitting sites in different scenarios; assigning staff members (as much as possible) to the same site regardless of the type of emergency; applying similar principles for storing and allocating life-saving and supplementary equipment; preparing the infrastructure to be utilized in different crises; and integrating generic modules in the training programs for the different hazards.

As Israel has had to deal with numerous types of emergencies in the past ten years, including MCEs, man-made conflicts, and pandemics, its experience may shed light on the effectiveness of the all-hazards approach and contribute to other decision makers in setting the policy regarding emergency management.

The aim of this study was to investigate implementation of the all-hazards approach in order to identify: 1) if preparedness of hospitals to a specific emergency scenario relates to preparedness for other types of emergencies; and 2) relationships between specific components to the overall preparedness for various emergencies.

## Methods

### Utilization of an evaluation tool to measure level of emergency preparedness

An evaluation tool consisting of 490 measurable and objective parameters was developed through a comprehensive literature review and recommendations of content experts. Of the 490 parameters, 239 were common for all emergency scenarios; the additional 251 were scenario-specific to mass casualty events, mass toxicological events, or biological events (67, 78, and 106 parameters, respectively). The content validity of the evaluation tool and the rate of importance of each parameter were determined through a modified Delphi process that included 229 content experts. Only parameters that were agreed upon by over 60% of the experts were included in the evaluation tool. The tool was tested in a pilot study conducted in two hospitals and, following its modification, evaluations were carried out by surveyors from the Ministry of Health and the Home Front Command. The evaluation process involved a review of the Standard Operating Procedures followed by a site visit during which all other components of the emergency preparedness were observed and measured. The overall score of readiness for emergencies was calculated utilizing a computer program that was specifically developed for this purpose, taking into account the level of importance of each parameter. The evaluation tool and its development were previously described [21] and were utilized to evaluate the level of emergency preparedness of all acute-care hospitals in Israel.

The 490 parameters, encompassing the various components of emergency preparedness, were classified into the following four categories:

1) Standard operating procedures (SOP) – based on national guidelines that were developed by the Ministry of Health (MOH), each hospital is required to develop its own SOP for the various hazards;

2) Training and drills – according to the policy set by the MOH, each hospital is required to conduct specific training programs and participate in both table-top exercises and full scale annual drills;

3) Knowledge of staff – the level of required knowledge of staff regarding the different components of emergency response to the various hazards is determined by the MOH;

4) Infrastructure and equipment – designated equipment essential for managing different hazards, such as ventilation machines, personal protective gear, vaccinations, and anti-viral drugs, must be procured to assure an effective emergency response. Similarly, vital infrastructure, such as decontamination sites or helipads, must be installed.

An example of parameters in each of the categories is presented in Table 1.

**Table 1:** Examples of parameters in each of the four categories

**Number**	**Category**	**Type of emergency**	**Parameter**
1	Standard Operating Procedures (SOPs)	Mass casualty event	The SOP for mass casualty events is updated for the last year
		Mass toxicological event	The hospital identified a decontamination team that will be deployed to the immediate site
		Biological event	The biological SOP includes a section regarding treatment of medical bio-hazard waste
2	Training & drills	Mass casualty event	80% of the surgical staff reinforcing the Emergency Department are graduates of an ATLS (advanced trauma life support) course
		Mass toxicological event	70% of the emergency department staff participated in a designated mass toxicological event training program
		Biological event	The hospital defined the medical staff that are required to participate in the biological training program
3	Knowledge of staff	Mass casualty event	The "nurse in charge" is proficient in using the public address system
		Mass toxicological event	More than 85% of the emergency department's nursing staff passed a toxicological test with scores >90
		Biological event	The emergency department physicians are proficient in the mechanism of sending samples to the microbiology laboratory
4	Infrastructure & equipment	Mass casualty event	The "immediate site" for treating severe casualties is equipped with a cart designated for treating children
		Mass toxicological event	At least 15 personal protection masks are immediately available in the emergency department
		Biological event	Designated sites for isolating patients suffering from infectious diseases have been defined

In addition to the evaluation tool, a random sample of approximately 30 physicians and nurses in each of the hospitals was given 56 standardized oral questions to evaluate their knowledge of the emergency preparedness process.

### Rating impact of each parameter on level of emergency preparedness

The parameters were rated by the experts according to three levels of importance. Level A indicated parameters with a high impact on emergency preparedness (representing 60% of the total preparedness score). Level B indicated parameters with a medium impact (representing 30% of the total score). Level C indicated parameters with the lowest impact (representing 10% of the total score). Mean rating scores for each parameter were calculated. The parameters were then classified according to four categories of emergency preparedness: SOPs, training and drills, infrastructure and equipment, and knowledge of staff.

### Evaluating levels of emergency preparedness in hospitals

The level of emergency preparedness was evaluated in 24 general hospitals in Israel, utilizing the evaluation tool by a team of 16 professional experts in emergency management from the MOH and the Home Front Command (HFC). Each component was evaluated by at least two raters, independently, and at the end of the evaluation process, the inter-rater reliability was calculated by comparing the findings of the two raters.

The evaluation process involved two phases: 1) an evaluation of the SOPs of the hospitals for MCE, MTE, and biological events prior to a site visit; and 2) a site visit by an evaluation team, at which time the remaining components for emergency preparedness were evaluated.

### Comparing levels of emergency preparedness to the various scenarios

The results of the hospital evaluations for the different scenarios were analyzed using an in-house computer program that was developed specifically to calculate a score for the level of preparedness. This score was calculated by multiplying the level of performance identified for each parameter (satisfactory performance allotted the maximal points; needing minor revisions allotted 70%; needing major revisions allotted 30%; while unsatisfactory performance allotted 0 points) by the relative value of the parameter (each Level A parameter was worth 0.57% of the overall score; each Level B parameter was worth 0.17%; and each Level C was worth 0.05% of the overall score). The points achieved for each parameter were summed in order to calculate the overall score of the hospital. The score for the level of emergency preparedness for each scenario was also analyzed in relation to each category: SOPs, training and drills, infrastructure and equipment, and knowledge of personnel.

### Statistical analysis

Data were analyzed using SPSS 15 (SPSS Inc., 2006). The preparedness scores of the hospitals were analyzed using the Spearman rho correlation coefficient, as follows: 1) Correlation of preparedness score for the different emergency scenarios (MCE, MTE, and biological events); 2) Correlation of scores in each category to the total preparedness score for the different emergency scenarios.

Correlation coefficients were defined as follows: rho = 0.25–0.44 – weak correlation; rho = 0.45–0.60 – moderate correlation; rho 0.61–0.80 – strong correlation, and 0.81–1.00 very strong correlation. Each level of correlation is regarded as statistically meaningful if *p* < 0.05.

## Results

### Relationship between overall hospital preparedness score for different emergency scenarios

**Figure 1:**
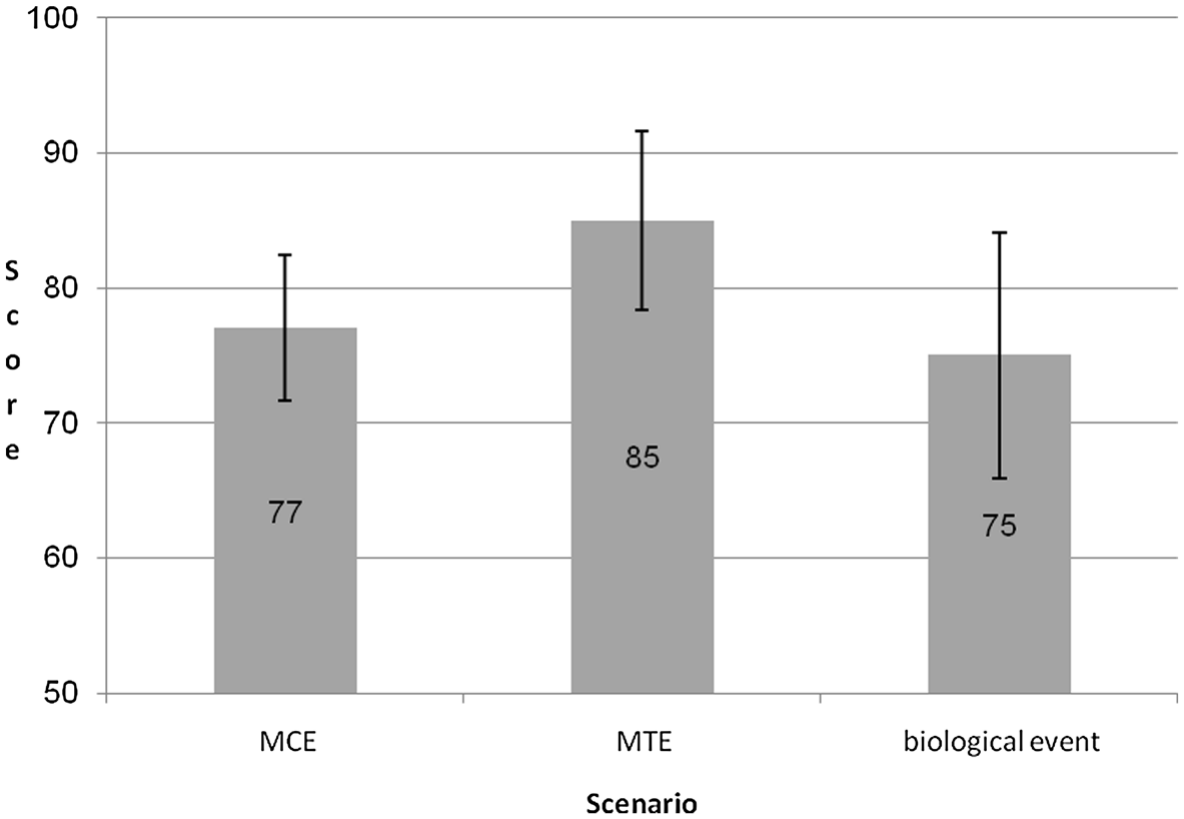
**Average overall preparedness scores of all general hospitals for different emergency scenarios**.

The overall preparedness scores of the hospitals for MCE, MTE, and biological events ranged from 32 to 100. The average preparedness scores and standard deviations for the different types of emergency scenarios are presented in Figure 1. Inter-rater reliability was high, ranging from 95.3% to 99.2%.

Medium relationships were found between the preparedness scores of the hospitals for the different emergency scenarios, as follows: 1) between MCE and MTE (.548, *p* = 0.006); 2) between MCE and a biological event (.541, *p* = .009); and between MTE and a biological event (.458, *p* = .032).

### Relationships between categories to total hospital preparedness score for the various emergency scenarios

Table 2 presents the correlations between preparedness of specific categories for each scenario with both overall preparedness for that scenario and overall preparedness for other emergency scenarios.

**Table 2:** Relationship between specific components and the total emergency preparedness score for different emergency scenarios

**Category**	**Emergency preparedness to MCE**	**Emergency preparedness to MTE**	**Emergency preparedness to biological event**
SOP			
SOPs for MCE	*p* > .05	rho = .581	*p* > .05
		*p* = .05	
SOPs for MTE	*p* > .05	rho = .667	*p* > .05
		*p* = .001	
SOPs for biological event	rho = .436	rho = .480	rho = .854
	*p* = .048	*p* = .028	*p* = .000
Training/Drills			
Training & drills for MCE	rho = .702	rho = .539	rho = .572
	*p* = .000	*p* = .012	*p* = .007
Training & drills for MTE	rho = .519	rho = .844	rho = .524
	*p* = .019	*p* = .000	*p* = .018
Training & drills for biological events	rho = .516	rho = .437	rho = .934
	*p* = .017	*p* = .047	*p* = .000
Knowledge			
Knowledge of personnel for MCE	rho = .650	rho = .619	rho = .533
	*p* = .001	*p* = .003	*p* = .013
Knowledge of personnel for MTE	*p* > .05	*p* > .05	*p* > .05
Knowledge of personnel for biological events	rho = .439	*p* > .05	*p* > .05
	*p* = .047		
Infrastructure			
Infrastructure & equipment for MCE	*p* > .05	*p* > .05	*p* > .05
Infrastructure & equipment for MTE	*p* > .05	rho = .749	*p* > .05
		*p* = .000	
Infrastructure & equipment for biological events	rho = .509	N/S	rho = .586
	*p* = .019		*p* = .05

### SOPs

Surprisingly, preparedness of SOPs for MCE was not found to be related to overall preparedness for that same scenario – MCE. Equally surprisingly, SOPs for MCE were found to be moderately related to preparedness for a different scenario – MTE. Preparedness of SOPs for MTE strongly correlated with preparedness for MTE, but did not correlate with preparedness for other emergency scenarios. Preparedness of SOPs for biological events strongly correlated with preparedness for a biological event, moderately correlated with preparedness for MTE, and weakly correlated with MCEs.

### Training and drills

A strong to very strong relationship was found between training and drills and the total preparedness score for the emergency scenarios. Regardless of the type of training and drills that were conducted, their scores correlated not only with preparedness for the same specific type of emergency scenario, but also with preparedness for other types of emergency scenarios. The levels of correlations among training and drills to the various types of emergencies are presented in Table 2.

### Knowledge of staff

Knowledge of healthcare personnel regarding MCE correlated with preparedness for all emergency scenarios: MCE, MTE, and a biological event. Knowledge of personnel regarding a biological event correlated only with preparedness for an MCE.

### Infrastructure and equipment

No significant relationships were found between infrastructure and equipment for MCE and preparedness for different emergency scenarios. Infrastructure and equipment for MTE was strongly related with preparedness for MTE. A moderate relationship was found between infrastructure and equipment for a biological event and preparedness for both MCE and biological events.

## Discussion

Based on theory alone and research prior to this study, one would not be able to conclude that preparedness for one scenario would enhance preparedness for other types of scenarios. At the same time, theory would suggest that there might be common components in the preparedness process for different emergency scenarios [8,20]. Given the fact that the process of preparing healthcare professionals to manage emergencies is both complicated and costly, it is important to optimize the emergency preparedness program by investing resources in the common components that may improve preparedness for different emergency scenarios [20,22]. The all-hazards approach provides a standardized approach for emergency preparedness, while still tending to hazard-specific components [23].

### Relationship between preparedness for different emergency scenarios

This study has shown that preparedness to a specific emergency is related to preparedness for other types of emergencies, thus strengthening the adoption of an all-hazards policy [2,9]. It appears that when steps are carried out to prepare for one emergency scenario, this also influences the ability of the hospital to prepare for other types of scenarios. Nevertheless, there is a need to explore to what degree preparedness to one emergency enhances preparedness to other scenarios.

### Relationships among specific categories with the total preparedness score for different emergency scenarios

It is a well-accepted assumption that a standard operating procedure is an essential requirement for emergency preparedness [2]. The findings of this study suggest that preparing an SOP might be especially important for scenarios for which hospital personnel are less well prepared or experienced [24]. A well-trained and experienced hospital staff may need to rely less on an SOP when dealing with a familiar emergency scenario, while in uncommon emergency scenarios, when the roles and expectations are less well known (as in mass toxicological events), a well-developed SOP is vital.

The relationship between knowledge related to MCE and preparedness for different types of scenarios would seem to suggest that there is a common hub of knowledge relevant to various emergency scenarios. The principles for managing an MCE seem to serve as the basis for other types of emergency preparedness programs [2], although scenario-specific knowledge must also be provided.

Training personnel and conducting drills are important factors of the emergency preparedness process in all scenarios [16]. Without the preparation of an SOP, provision of knowledge and capabilities to healthcare personnel, effective training, and drills, it is not possible for a hospital to achieve emergency preparedness [14,17]. This assumption was strongly supported by the findings of this study.

Infrastructure and equipment were found to relate to the total preparedness score for an MTE and a biological event. This most probably is derived from the unique requirements that are specific to these scenarios, such as personal protective gear. Given that hospital staff is likely to be unfamiliar with this equipment and infrastructure, it is necessary for training programs to include opportunities for staff to become adept in their use. In contrast, infrastructure and equipment required for MCEs are similar to what is routinely utilized in the emergency department, and thus staff is well acquainted with them. This might explain the lack of correlation between infrastructure and equipment with preparedness for an MCE.

## Limitations

Preparedness for radiological events in Israel is based on the doctrine of admitting and treating toxicological casualties. A unique designated doctrine is relevant only to seven referral hospitals. Therefore, the evaluation of preparedness of this specific hazard was not included in the study.

This study does not provide an answer to the extremely important question of how often exercises and drills need to be held in order to assure retention of knowledge and competencies. This should be further investigated, as there tends to be an attrition of knowledge and skills fairly rapidly amongst staff who are not routinely actively involved in emergency management. While the findings of this study present the relationship between hospitals' preparedness for different types of emergencies, the study does not address the degree that preparedness for one type of emergency actually enhances preparedness for other emergency scenarios. Rather than reflecting causal relationships in preparedness across different types of emergencies, it might be that these associations reflect a common causal factor, such as strong leadership and/or strong commitment of the hospital's management towards assuring effective emergency preparedness and response.

## Conclusions

The findings of this study present the relationship between preparedness for different emergency scenarios. There are fundamental critical building blocks such as SOPs, training, and drills programs that improve preparedness for different types of emergencies more than other building blocks, such as equipment or knowledge of personnel. Investing efforts in promoting those components of the preparedness for one scenario may contribute to improved preparedness for other scenarios.

SOPs appear to be an important element in achieving emergency preparedness, especially for emerging scenarios, while knowledge is the basis for managing familiar emergencies. Policy makers should identify the knowledge and skills that are relevant for different types of scenarios, and emphasize them in the training programs.

The findings of this study provide evidence-based support for the all-hazards approach to emergency preparedness, particularly with regard to standard operating procedures, training, and drills. Policy makers in the field of emergency management should discourage the healthcare systems from developing unique designated plans for each type of emergency scenario, but rather focus on identification of similar characteristics of various crisis situations and invest efforts and resources on preparing those components.

## Competing interests

None of the authors had (nor have) competing interests.

## Author contributions

BA designed the study, analyzed the data and drafted the article. AG reviewed the manuscript and provided continuous supervision over the design of the study. RC analyzed the data, edited the manuscript and reviewed it. DL was instrumental in conducting the study. YBD reviewed the manuscript and provided continuous supervision over the design of the study. All authors read and approved the final manuscript.

## Funding

No internal or external funding sources were allocated.
